# Does the medical insurance system really achieved the effect of poverty alleviation for the middle-aged and elderly people in China? Characteristics of vulnerable groups and failure links

**DOI:** 10.1186/s12889-020-08554-3

**Published:** 2020-04-03

**Authors:** Meiyan Ma, Ye Li, Nianshi Wang, Qunhong Wu, Linghan Shan, Mingli Jiao, Xuelian Fu, Heng Li, Tao Sun, Bin Yi, Wanxin Tian, Qi Xia, Baoguo Shi, Yanhua Hao, Hui Yin, Ning Ning, Lijun Gao, Libo Liang, Jiahui Wang

**Affiliations:** 1 Department of Social Medicine, School of Health Management, Harbin Medical University, Harbin, 150086 Heilongjiang China; 2 The Second Affiliated Hospital of Harbin Medical University, Harbin, Heilongjiang China; 3 China Hospital Development institute of Shanghai Jiao Tong University, Shanghai, China; 4 Department of Health Service Management, School of Medicine, Hang Zhou Normal University, Zhejiang, China; 5 The First Specialized Hospital of Harbin, Harbin, Heilongjiang China; 6 Department of Economics, School of Economics, Minzu University of China, Beijing, China

**Keywords:** Medical insurance, Poverty alleviation, Healthy poverty, Catastrophic health expenditure, Impoverishment by medical expenses

## Abstract

**Background:**

We examined the physiological, household, and spatial agglomeration characteristics of the health poverty population in China. We identified weak links that affect the implementation of the medical insurance and further improve its effectiveness for health poverty alleviation.

**Methods:**

A national representative sample from the China Health and Retirement Longitudinal Study (CHARLS) was analyzed. The WHO recommended method was adopted to calculate catastrophic health expenditure (CHE) and impoverishment by medical expenses (IME). We created a binary indicator for IME as the outcome variable and applied the treatment-effect model to analyze the determinants of IME.

**Results:**

The incidence of IME was 7.2% of the overall population, compared to 20.3% of the sample households trapped in CHE. The incidence of IME enrolled in insurance schemes was 7.4% higher than that of uninsured families (4.8%). Economic level, living area, family size, age of household head, having hospitalized members, and participating in insurance were statistically significant for the occurrence of IME.

**Conclusions:**

The original poverty-promoting policies has not reached the maximum point of convergence with China’s current demand for health. The overlapped health vulnerabilities exacerbated the risk of poverty among the elderly and households with high health needs and utilization. In addition, the medical insurance schemes have proven to be insufficient for protection against economic burden of poor households. So, special health needs, age, and household capacity to pay should be comprehensively considered while strengthening the connection between the disease insurance scheme with supplementary insurance.

## Background

Poverty continues to be a worldwide problem, as of 2015, there are still more than 10% of the world’s population lived on less than US$1.90 a day [1, 2]. Measurement of poverty has gradually changed from a single economic dimension to multiple dimensions integrating economic, cultural, health, and dignity indicators and some scholars define poverty as a lack of basic needs, health, and social capital [3, 4]. People who fall into healthy poverty often cannot access health services, which affects their health level, and ultimately their ability to obtain income. The poverty then worsens and they may easily become trapped in a cycle of “poverty-health deterioration-more poverty” [5].In India, health deprivation is second only to economic income in terms of factors leading to high poverty rates [6]. True health poverty means both the loss of health ability and the deprivation of social health rights.

Since the beginning of reform and opening up in 1978, China has significantly alleviated its poverty. Between 1978 and 2018, the poor population decreased from 770 million to 16.6 million [7].Moreover, the global population of extreme poverty fell from 1.9 billion in 1990 to 836 million in 2015,and in this process, China’s contribution to poverty reduction exceeded 70% [8]. However, with changes to China’s population structure and chronic disease-based disease spectrum, traditional poverty alleviation methods cannot accurately identify the characteristics of the poor, causing bottlenecks in poverty alleviation. From 1978 to 1999, China reduced poverty at a rate of 1.5% per year. However, in the following 8 years, this rate was only approximately 0.26% [9]. In 2017,approximately 45% of poor people are impoverished due to major illness and disabilities and 260 million people in China have chronic diseases, causing approximately 70% of the disease burden [10, 11]. Additionally, the prevalence of chronic disease in the elderly is 2.9 times that of the total population [12].The inherent weakness of elderly who are vulnerable to suffer from chronic diseases, not only lead to the shortage of labor, but also increase demand for health services,which all contribute to more economic risks and health poverty.

To reduce the disease burden, medical insurance has played a certain role in economic protection, China’s basic medical insurance system consists of three types of medical insurance scheme: the Medical Insurance for Urban Employees (MIUE) is designed for cost sharing between employers and employees, with risk polling managed at the municipal level. The Medical Insurance for Urban Residents (MIUR) is for urban residents who are not covered by MIUE and is co-financed by enrollees and local government. The New Cooperative Medical Scheme (NCMS) is designed for rural residents covering mostly inpatient services and a few outpatient services [13]. The coverage of the basic medical insurance schemes has achieved the breath of universal coverage with participation rate of 97%.

However, studies have demonstrated that the actual effect of this coverage has been offset by the rapid escalation of medical expenses [14]. In 2012, the proportion of Chinese residents’ Out-of-Pocket (OOP) expenses to total health expenditure was 35.69%, 2.3 times higher than the international average (15.28%) [15]. The WHO (World Health Organization)2017 Global UHC Report demonstrates that 17.7% of Chinese people spend more than 10% of their household budget on OOP medical payments, far higher than OECD countries [16]. The WHO proposes a reasonable OOP proportion of 15%~ 20%, but as of 2017, the ratio in Chinese residents is as high as 28.77% [17]. Although previous studies have shown that participating in medical insurance is conducive to reducing the risk of catastrophic health expenditures (CHE) in the family, and it can reduce the basic economic burden of the family. However, under the background of the reform of the medical insurance system, there are still many people in China who have a heavy OOP burden. In China,there are still more than 34 million poor people, of which 33% are due to lack of labor caused by disease, and 12% are due to CHE and high medical expenses [10].Kumar K found that, the OOP’s share in total expenditure in China was 15.1%,much higher than India (11.5%) [18].In addition, numerous studies have shown that,high medical expenses increase the risk of poverty. E.g,Ravi et al. estimated that, OOP health expenses increased hidden poverty’s rate by 7.5 points in India [13, 19, 20].

Especially in China where OOP accounting for a relatively high proportion, we found that,the OOP by residents are the key factors affecting the occurrence of impoverishment by medical expense (IME). It can be seen that, previous poverty alleviation policies concentrated on accelerating China’s poverty reduction through economic construction, education, and human capital investment to reduce material poverty. The traditional poverty alleviation policy is no longer suitable for China’s current poverty-stricken conditions, nor can it precisely target the most vulnerable people who are poor due to disease. Improvement in the health of the population can reduce the occurrence of poverty [21]. Both China and international organizations have emphasized that, the health poverty alleviation is of great significance to guaranteeing the poor population access to basic medical and health services, and preventing “disease from poverty” and “return to poverty from disease”, and has become a promotion key nodes in poverty reduction [22, 23].So, China must change the way of poverty alleviation only through economic means and make health poverty the focus.

The introduction of the “Healthy China Project” in 2002, firstly shifted people’s poverty reduction focus to a healthy perspective, but only gave a framework for action [24]. With the promulgation of the targeted poverty alleviation policy in 2016, healthy poverty alleviation has been taken as an absolute area of priority intervention. The accurate identification of health poverty population contains two approaches: firstly, it identifies the poor people and household by the national poverty alleviation standards, and selects “impoverishment households by medical expense” and “health poverty population” from all the poor people and poor households who set up files according to the household surveys. Secondly, it identifies health poverty population by a certain comprehensive standards, that is, classify “the people who are likely to be poor due to illness” and “vulnerable groups” into the health poverty alleviation targets. The main object of health poverty alleviation is rural poor population. The key scope of health poverty alleviation is poor areas [25, 26].With the implementation of health poverty alleviation policies, the government conducts series of actions to achieve convergence of basic medical insurance, major disease insurance, medical assistance and emergency medical assistance. However, as a basic institutional means of health poverty alleviation, the medical insurance system has only achieved population width coverage with almost 95% of the population having access to health services. A study from China has shown that, the annual medical spending of depression has reached to nearly $42.67 per person [27],but the welfare package of the medical insurance only covers 33 kinds of drugs for mental disorders [28].Moreover, in 2018, the actual rate of reimbursement for NCMS hospitalization was only 50%, far lower than the 75% required by the policy [29].And the OOP of Chinese accounted for 30%, which is much higher than the national average of the WHO member countries (18.6%) [30]. The depth (welfare package coverage) and height (compensation level) of medical insurance are still need to be strengthened, so as to effectively play to the economic protection role for poverty alleviation system. In short, although China has gradually implemented some healthy poverty alleviation plans, there are still many weak links, such as, lacking precise and quantitative identification mechanism of marginal groups that are prone to fall into poverty due to illness and lacking assistance programs for vulnerable groups (the elderly, severely disabled people, people suffering sudden catastrophic medical expense) [31]. How to give full play to the basic role of the medical insurance system in precise poverty alleviation policies? It is the key to achieving health poverty reduction.

Based on the perspective of healthy poverty, we adopt the WHO recommended method to verify the health-reducing effect of the health care system, that is, whether the medical insurance system reduces the risk of IME among the middle-aged and elderly people. In addition,we also analyze the physiological, household, and spatial agglomeration characteristics of the health poverty population and scientifically identify groups at high risk of health poverty. It is important to understand whether medical insurance system increases the accessibility and affordability of health care, which provides good evidence for further ways to alleviate health poverty.

## Methods

### Data source and sampling method

The data used to calculate the rates of IME and CHE were obtained from the 2015 China Health and Retirement Longitudinal Study (CHARLS) database, which is designed to collect microscopic information from middle aged and elderly people over the age of 45. The filter questionnaire was used to determine whether the candidate has 45 or older respondents. If there were several households living in the sample address and more than one household had age-appropriate respondents, the system would randomly select one household to conduct the survey, and then list all the household members and their age. If there was only one age-appropriate respondent, he/she directly became the "main respondent". If there were multiple age-appropriate respondents, one was randomly selected as the "main respondent". The survey used four-stage stratified cluster sampling to select eligible individuals. First, all the counties in 28 provinces countrywide were sorted according to per capita GDP, and 150 counties (cities) were then randomly selected by PPS (probabilities proportionate to size as measured by population). Second, three villages or communities in each county were randomly selected and a data-set of 450 samples was generated. Then,80 households in each village or community were randomly selected. 21,101 samples were included in the survey. Finally, A total of 19,144 samples (9167 households) were finally obtained after cleaning the incomplete data and missing values.

### Data collection and quality control

Data were collected through questionnaires comprising the following eight parts: A. Household registration form, B. Demographic background, C. Family information, D. Health status and function, E. Health care and insurance, F. Work, retirement, and pension, G/H. Income, expenses, and assets. Face-to-face household interviews were conducted by qualified investigators. Quality control was implemented by supervisors and included GPS comparison, data verification, recording verification, and telephone verification. Based on the research needs of IME, we largely focused on demographic information, health status, medical insurance, income, and expenditure.

### Statistical analysis

#### Calculation method for CHE and impoverishment by medical expense (IME)

We adopted the WHO recommended method to calculate CHE and IME. CHE was defined as an OOP payment for health care equaling or exceeding 40% of a household’s capacity to pay [32]. IME was defined as consumption expenditure equal to or higher than household subsistence expenditure but lower than the subsistence expenditure (SE) net of OOP health payments. The key expenditure indicators involved in the calculation process are as follows:

#### Out-of-pocket health expenditure (OOP)

The payments made by households for their health services without third-party compensation; Household consumption expenditure (exp) comprises both monetary and in-kind payment on all goods and services, and the money value of the consumption of home-made products. Household subsistence expenditure (SE) was calculated using food expenditure as a share of total household consumption expenditure. The weighted average food expenditure of a household, whose food expenditure as a share of household consumption expenditure fell between the 45th and 55th percentiles of the entire sample, was treated as the poverty line. The SE of each household was calculated as the poverty line multiplied by the standard household size. A household’s capacity to pay (CTP) was defined as non-subsistence spending of a household as a share of total household consumption expenditure.

#### Treatment effect model and instrumental variables

The relationship between participation in medical insurance system and IME is characterized by a joint causality. Participation in medical insurance system exists hidden selection bias, unobserved characteristics such as self-selection of participation, self-assessment health status are correlated with initial treatment choice. To address the bias resulting from hidden selection bias and joint causality, we applied an instrumental variable (IV) approach known as the treatment-effect model [33, 34].

#### Instrument indicators

We applied the treatment-effect model to instrument the indicators of participation in medical insurance system, that is to say, participation in medical insurance system is endogenous. We need to find instrument variables which are related with endogenous predictors (participation in medical insurance system) but not related to the error term of outcome variables (IME) [35].For the further identify whether endogenous variables existing in the regression equation, we use DWH (Durbin-Wu-Hausman) test to examines whether the endogenous predictor is truly endogenous (The heterogeneity test result shows that, *P* = 0.0224 < 0.05, so we use the DWH test). And the DWH test,shows, *P* = 0.0450 < 0.05,The results confirm that there is indeed endogeneity in our model.

In order to identify good instruments, we first reviewed the existing literatures. We identified two potential instruments: the self-assessment health status and the average community participation rate of medical health insurance schemes. Generally speaking, self-reported groups with poor health (whether poor or rich) are more likely to choose to participate in medical insurance to protect their health [36].In additional, the community participation rate of basic medical insurance for residents has an important impact on the willingness of households to participate in the insurance. The people living in the same community has certain common characteristics such as the household economic level and health management awareness, which all contribute to their participation to medical insurance. Moreover, some of the basic medical insurance schemes in China are community-based units. For example, NCMS and MIUR use the community as a promotion unit, and the village cadre mobilize the villagers to participate in the medical insurance schemes, which makes the high correlation between the community participate rate and the household participation willingness [37, 38].

Good instrument should satisfy two main criteria known as relevance and validity criteria, that is to say, good instruments would be correlated with the endogenous variable (relevance criteria) but not to be correlated with the error terms in the model of the outcome variable (validity criteria).

We use the Over-identification Test and *F* value in GMM regression to check the validity and relevance [39, 40]. In addition, in order to further investigate the weak tool variable problem, we also perform redundancy testing. The results show that community participation rate becomes our ultimate effective instrumental variable. The results regarding the validity and relevance are **presented in** Table 1.

**Table 1:** Validity test and correlation test presented of people

Instrumental variable	DWH Test	Over-identification Test	F Test	Redundancy Test
Community participation rate	chi2 [1]=4.0201 (*p* = 0.0450 < 0.05)*	chi2 [1]=0.1053 (*p* = 0.7455 > 0.05)*	*F* = 35.9921 > 10	Chi-sq [1] =0.0000*
Self-assessment health				Chi-sq [1] =0.6096

#### Outcome variable and covariates

We created a binary indicator for IME as the outcome variable (1 = occurrence, 0 = no occurrence). Based on a large literature review, variables on social-demographic characteristic (age, gender, occupation, marital status, education level of household head, family size, presence of over 65-year-olds) and health service needs and utilization (having hospitalization members, chronic patients, hospitalization rate, and non-admission rate (defined as the percentage of patients requiring hospitalization but who were not hospitalized) were used as control variables. According to the WHO classification criteria, 14 types of chronic disease in the questionnaire were reclassified into six categories: cardiovascular, respiratory, mental, cancer, diabetes, and other chronic diseases.

The treatment-effect model consists of a two-stage regression. In the first stage, we regress the outcome variable on the covariates. Based on the results of the first phase, we add instrument variables and perform a quadratic regression on the outcome variable.

## Results

### Basic information

The total sample comprised 9167 households and 19,144 individuals, of which 52.5% of household heads were male, and up to 65.8% of the middle-aged and elderly people were < 64 years. Most had primary and junior high school education level (56.6%), and more than half (61.1%) of elderly members had chronic diseases. Finally, 92.6% had medical insurance (Table 2).

**Table 2:** Sample characteristics

Variables	Variable value	Percentage(%)	Mean	SD
**Outcome variable**	0 = No	92.8	0.07	0.259
	1 = Yes	7.2		
Participate in medical insurance scheme	0 = No	7.4	0.971	0.001
	1 = Yes	92.6		
Gender of household head	0 = Female	47.5	1.475	0.499
	1 = Male	52.5		
Marital status of household head	0 = Others	14.2	0.858	0.348
	1 = Married	85.7		
Education level of household head	1 = Illiteracy	18.9	1.923	0.590
	2 = Elementary to junior high	56.6		
	3 = High school and above	12.2		
Family size	1 = 1	61.3	1.447	0.607
	2 = 2–3	32.6		
	3 = More than 3 people	6.1		
Age of household head	1 = 45–54	33.3	2.080	0.968
	2 = 55–64	32.5		
	3 = 65–74	23.2		
	4= > 75	9.0		
Having members over 65 years old	0 = No	65.8	1.671	0.469
	1 = Yes	32.3		
Occupation of household head	1 = Agriculture	27.4	1.551	0.807
	2 = Employed	10.2		
	3 = Self employed	4.7		
	4 = Free	1.2		
	5 = Others	4.6		
	6 = Retirement	9.5		
	7 = Unemployed	7.3		
Having hospitalization members	0 = No	86.2	0.131	0.337
	1 = Yes	13.0		
Having NCD members	0 = No	38.9	0.611	0.487
	1 = Yes	61.1		
Household consumption per capita quintile	Lowest	20.6	2.963	1.410
	2	20.4		
	3	20.1		
	4	19.8		
	Highest	19.1		
Region	1 = Eastern	34.9	1.874	0.746
	2 = Middle	42.7		
	3 = Western	22.3		
Urban and rural	0 = Rural	79.0	0.209	0.406
	1 = Urban	20.8		

### Health-care needs and service utilization

The overall monthly prevalence of the surveyed sample was 11.8%, chronic disease was 60.7%, hospitalization rate 13.5%, and hospitalization reimbursement ratio 49.7%. These indicators demonstrate different trends for different populations as follows (Table 3):

**Table 3:** Health-care needs and service utilization

		Prevalence (%)	Chronic disease prevalence (%)	Hospitalization rate (%)	Non-admission rate (%)	Hospitalization reimbursement ratio (%)
Family size	1	15.9	63.2	13.4	5.9	39.3
	2–3	14.0	61.8	13.8	5.9	49.9
	> 3	14.2	58.6	12.9	5.5	55.1
Hospitalization members	Yes	22.5	65.7	100.0	/	49.7
	No	13.1	60.0	0.0	4.8	/
Disabled members	Yes	20.1	63.7	15.6	9.0	56
	No	13.9	59.8	13.4	5.5	50.9
Chronic disease	Respiratory diseases	17.4	/	16.7	7.6	53.3
	Cardiovascular diseases	13.9	/	14.9	6.4	50.2
	Mental illness	14.8	/	17.7	6.3	54.9
	Diabetes	11.5	/	15.2	5.9	62.9
	Cancer	18.5	/	9.7	6.2	57.4
	Others	15.9	/	14.8	6.8	47.5
Medical insurance schemes	MIUE	11.8	57%	16.4	4.3	65.8
	MIUR	14.6	60.1	16	6.5	58.7
	NCMS	14.6	61.8	13	6.1	42.2
	Integrated insurance	16.8	57.7	12.5	4.4	34.1
	Others and no have insurance	12.6	58.1	15.7	5.7	48.5
Economic quintile	Lowest	14.5	61.0	13.4	5.9	49.9
	2	14.4	65.0	12.6	6.2	42
	3	14.1	61.0	12.5	5.5	59.6
	4	14.6	59.9	13.4	5.8	47.1
	Highest	13.4	56.7	15.8	5.5	50.5
Overall		11.8	60.7	13.5	5.8	49.7

[1] The population with high level of health service demand but low utilization of health services and low reimbursement rate. For example, respiratory disease members had a high demand for health services (prevalence rate 17.4%) but low level of health service utilization (no-admission rate 7.6%) and an adequate reimbursement level (53.3%). Additionally, the prevalence of families participating in integrated insurance (16.8%) and the prevalence of chronic diseases (57.7%) were significantly higher than for the MIUE (11.8 and 57%), but the hospitalization reimbursement ratio (42.2%) was 23.6% lower than for the MIUE (65.8%). People from different economic groups demonstrated a similar phenomenon.

[2] High health utilization but low reimbursement. Households with mental illness had a higher hospitalization rate (17.7%) but a hospitalization reimbursement ratio (54.9%) that was lower by nearly 8% than cancer households (62.9%). Patients who participated in MIUR demonstrated a similar pattern.

### Region level: poverty, catastrophic health expenditure, impoverishment by medical expense in different provinces

**Fig. 1:**
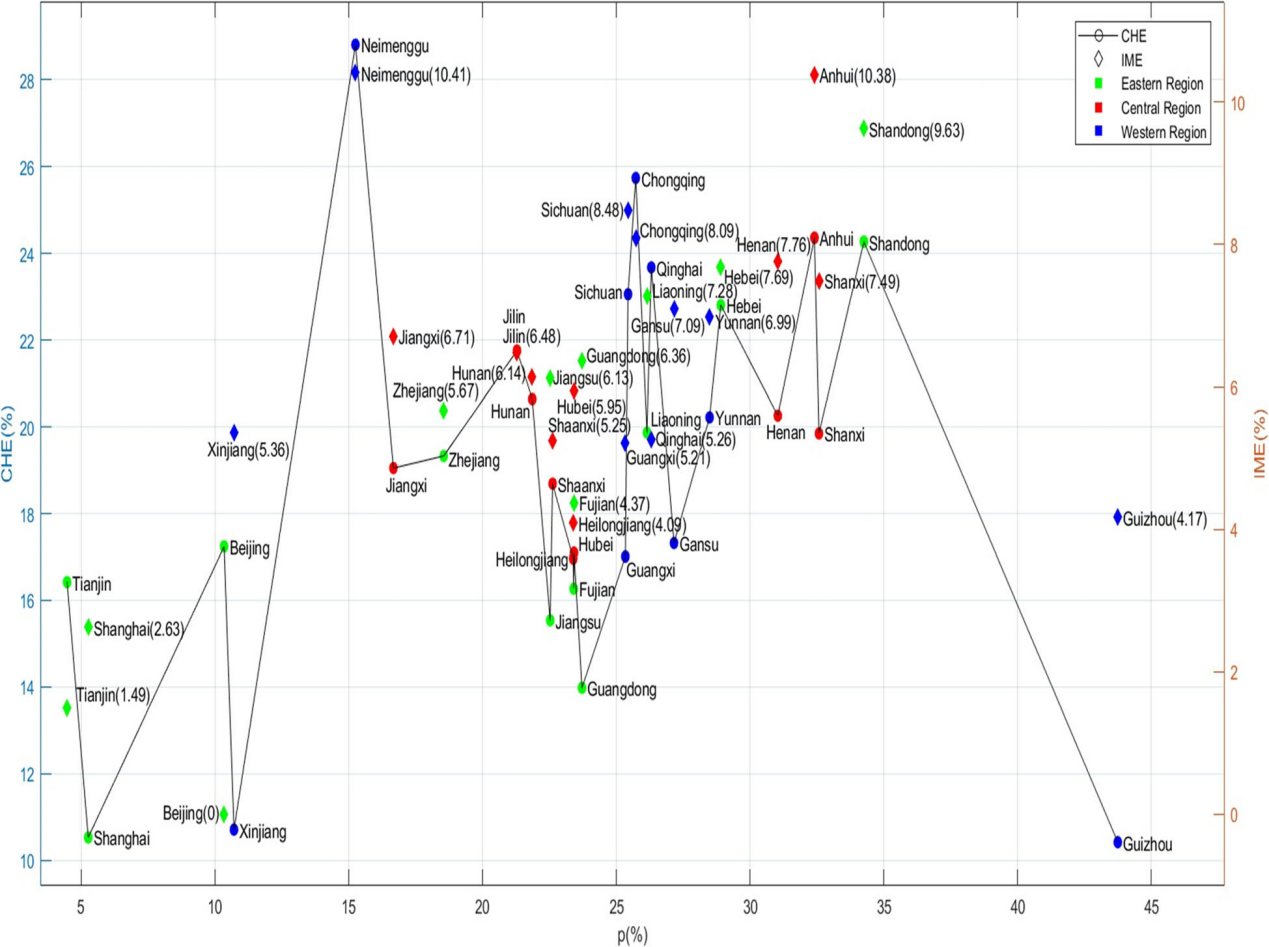
Poverty, catastrophic health expenditure, impoverishment by medical expense

The poverty rate ranges from 4~45%, with the highest rate in Guizhou (43.75%), which is approximately 10 times that in Tianjin (4.47%). As shown in the figure, poverty rates are generally higher in central (21–33%)and western provinces (22–29%)than in eastern provinces (18–25%).To verify the disease burden of middle-aged and older over 45, we also measured the incidence of IME and CHE in different provinces. Among them, Neimenggu (10.41%), Anhui (10.38%), Shandong (9.63%), Sichuan (8.48%), and Chongqing (8.09%) were the provinces most burdened by IME; In terms of CHE rate, the top five incidence rates are: Neimenggu (28.81%), Chongqing (25.73%), Anhui (24.36%), Shandong (24.26%), Qinghai (23.68%) (Fig. 1).

It is worth noting that, in the case of a similar level of poverty, residents’ utilization of health services caused significant differences in the incidence of CHE and IME: the levels of poverty in Chongqing, Guangxi, and Sichuan were originally equal, but after paying medical expenses, the incidence of CHE in Chongqing (25.74%) was higher than that in Guangxi and Sichuan. Moreover, the poverty rate in Guizhou (43.75%) was much higher than in Neimenggu (15.25%). However, after paying for health services, the risk of IME (10.41%) was twice as high as in Guizhou (4.17%).

### Family level: poverty, catastrophic health expenditures and related indicators

The muti-dimensional analysis on the incidence of CHE and IME in elder households demonstrated that the IME occurred in 7.2% of the overall population compared to 20.3% of sample households trapped in CHE. OOP accounted for 18.72% of family payment capacity and households enrolled in insurance schemes were more likely to suffer from IME (7.4%) and CHE (20.5%) than uninsured households (4.8 and 17.9%) (Table 4).

**Table 4:** Poverty, CHE, and IME in different households

		Incidence of IME (%)	Incidence of CHE (%)	OOP/CTP (%)	Out-of-pocket (yuan)	Capacity to pay (yuan)
Family size	1	8.1	23.0	21.1	336.7	1590.6
	2–3	5.7	16.1	15.3	397.3	2588.1
	> 3	6.2	15.8	14.8	348.1	2345
Age of household head	45–54	4.4	12.4	12.6	310.1	2460.5
	55–64	7.4	20.6	19.4	372.2	1910.6
	3 = 65–74	9.7	27.0	25.6	401.2	1564.1
	> 75	10.1	32.0	26.8	375.8	1399.2
Hospitalization members	Yes	9.9	29.3	31.5	640.2	2030.0
	No	6.8	19.0	16.2	315.6	1948.0
Medical insurance	MIUE	3.5	15.7	13.9	438.4	3133.2
	MIUR	5.6	15.8	16.4	377.1	2291.0
	NCMS	7.9	21.4	19.0	320.0	1676.6
	Integrated insurance	8.5	18.2	7.8	210.7	2674.5
	Others and no have insurance	4.8	17.9	22.7	601.0	2638.7
Household consumption per capita quintile	Lowest	8.0	19.4	19.0	315.6	1660.7
	2	8.3	22.6	21.5	382.5	1773.7
	3	7.6	20.9	19.4	347.1	1785.5
	4	6.6	19.2	17.3	331.1	1913.7
	Highest	5.3	19.4	15.1	412.3	2722.3
Participate in insurance	Yes	7.4	20.5	17.7	331.9	1873.2
	No	4.8	17.9	22.7	601.0	2638.7
Overall	/	7.2	20.3	18.2	357.1	1961.7

As the age of household head increased, the risk of households being trapped in IME also increased. The incidence of IME among the elderly > 75 years old (10.1%) was 2.2 times that of the 45–54 age group. Moreover, MIUE had the least risk of economic burden, and the incidence of IME was 3.5%, only half the total sample population (7.2%). The proportion of OOP to household payment capacity was 13.9%, significantly lower than MIUR (16.4%) and NCMS (19.0%). The incidence of IME in NCMS (7.9%) was equivalent to that of the integrated medical insurance family, but the burden of health expenditure (19.0%) was 2.1 times more. Meanwhile, except for the sub-poverty group, the incidence of IME decreased with the improvement of the economic quintile. The poorest group shared the highest IME rate (approx. 8.0%), which was 2.7 percentage points higher than the richest group (5.3%).

### Treatment-effect model analysis

The model demonstrated that economic levels, living area, family size, age of household head, having hospitalization members, and participating in insurance were statistically significant for IME (*P* < 0.05) (Table 5).

**Table 5:** Results of the Treatment-effect model

	Coef.	Std. Err.	z	***P*** > |z|	[95% Conf Interval]	
**IME**						
Gender of household head	0.0147	0.0078	1.87	0.061	−0.0006	0.0300
Education level of household head	0.0118	0.0070	1.69	0.091	−0.0018	0.0256
Marital status of household head	−0.0039	0.0108	−0.37	0.714	−0.0251	0.0172
**Household consumption per capita quintile**	**−0.0060**	**0.0028**	**−2.13**	**0.033**	**−0.0116**	**− 0.0004**
Region	0.0044	0.0052	0.85	0.395	− 0.0057	0.0146
**Urban and rural**	**−0.0280**	**0.0102**	**−2.73**	**0.006**	−.0481	−0.0079
Type of chronic disease	0.0528	0.0330	1.60	0.110	−.0119	0.1175
Occupation of household head	0.0002	0.0018	0.14	0.887	−0.0034	0.0039
**Family size**	**−0.0159**	**0.0064**	**−2.48**	**0.013**	**−0.0284**	**−0.0033**
**Age of household head**	**0.0183**	**0.0039**	**4.65**	**0.000**	**0.0106**	**0.0261**
**Having hospitalization members**	**0.0230**	**0.0113**	**2.04**	**0.041**	**0.0009**	**0.0452**
Cardiovascular diseases	−0.0453	0.0337	−1.34	0.180	−0.1115	0.0209
Chronic respiratory disease	−0.0482	0.0321	−1.50	0.134	−0.1113	0.0147
Other chronic diseases	−0.0589	0.0334	−1.76	0.078	−0.1245	0.0066
Cancer	−0.0375	0.0496	−0.76	0.450	−0.1348	0.0597
Diabetes	−0.0583	0.0315	−1.85	0.064	−0.1201	0.0034
Mental illness	0.0099	0.0366	0.27	0.787	−0.0619	0.0817
Having disabled members	0.0184	0.0215	0.85	0.393	−0.0238	0.0607
**Participate in insurance**	**0.1655**	**0.0831**	**1.99**	**0.047**	**0.0025**	**0.3285**

Economic level and family size with the middle-aged and elderly people demonstrated a negative correlation with incidence of IME: the family’s economic income rises by higher level, the risk of IME was 0.6%age points reduction (a similar phenomenon occurred family size); age was positively correlated with rate of IME, that is, as the age of the head of household increased, the risk of family impoverishment by medical expense increased by 1.8 percentage points. Households with hospitalization members increased the risk of IME and the risk of poverty was 2.3 percentage points, higher than for families without hospitalization members. The model results demonstrated that living in urban areas reduced the probability of IME more than living in rural areas. However, participation in medical insurance was more likely to cause IME, and increased the risk of being trapped in IME by 16.5 percentage points.

### Medical insurance level:factors affecting IME by different medical insurance systems

**Fig. 2:**
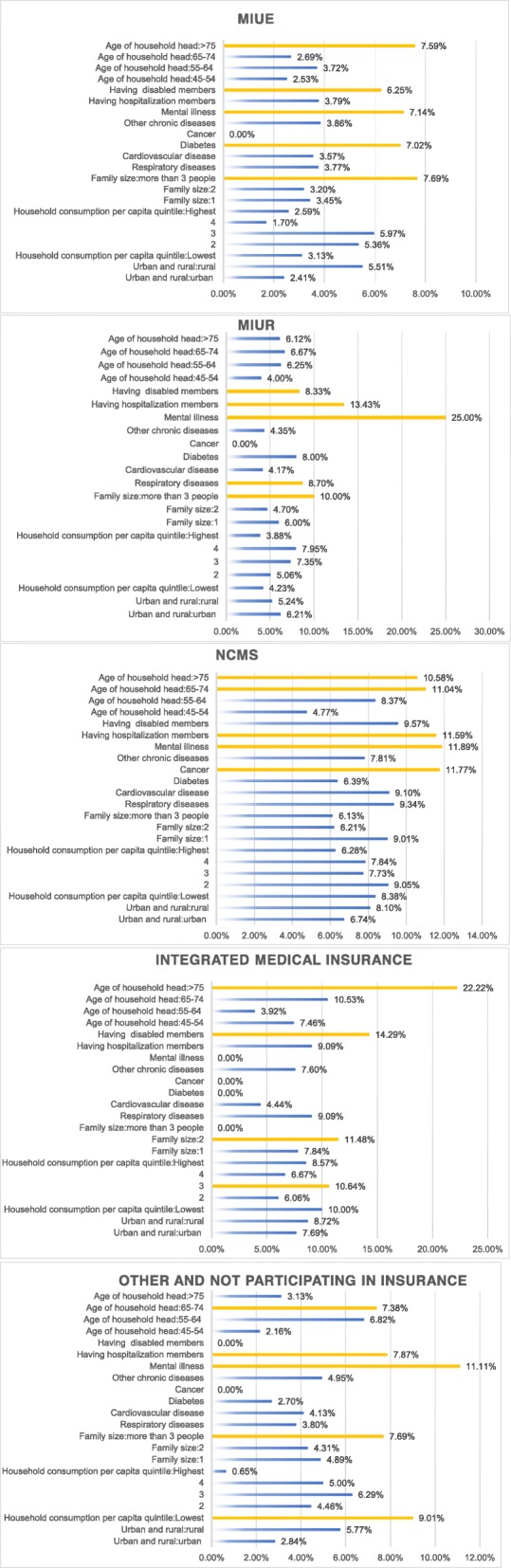
Factors affecting IME by different medical insurance systems

All the results indicated that people with medical insurance were more likely to suffer from IME. To identify the key bottlenecks, we conducted a series of calculations (seen in Fig. 2):

Households with mental illness, cancer, diabetes, hospitalization, and > 65-year-old members as well as those living in rural areas and of a lower economic level were at high risk of IME. For households enrolled in MIUR, NCMS, and households without medical insurance, having members with mental illness was the primary factor in impoverishment, with IME at 25.0, 11.89, and 11.11% respectively. The top five influencing factors of integrated medical insurance families demonstrated the highest rate of IME, generally above 10.64%. Having a household head > 75 years old was the first influencing factor (IME 22.22%), at 15 percentage points higher than the overall population (7.23%). For people enrolled in NCMS, the rate of the top five influencing factors ranged between 10 and 12%, second only to integrated medical insurance. Cancer was the only important factor affecting the IME of NCMS, with the incidence as high as 11.77%.

## Discussion

Since the 1990s, health poverty has been officially adopted as an important agenda worldwide [41].The Sustainable Development Goals (SDGs) clearly demonstrate that health protection policies should be developed for the most vulnerable populations [42].In 2016, “Guiding Opinions on Implementing a Healthy Poverty Alleviation Project” clearly stated that the Health Poverty Alleviation Project was key to preventing IME [43].

The rate of CHE for middle-aged elderly people in China (20.3%) was higher than in other low and middle income countries. Amaya-Lara found that,9.6% of Colombian households had catastrophic expenditure [44].In India, the incidence of catastrophic health expenditure for older people over 65 was only 7%, much lower than in China [45].And in Iran,the rate of catastrophic health expenditure headcount ratio varied from 0.5 to 14.3% and from 0.48 to 13.27% for rural and urban households, respectively [46]. The incidences of IME (7.4%) and CHE (20.5%) participating in medical insurance were 2.6% higher than those of uninsured households (4.8 and 17.9%). Our research also found that families with mental illness, elderly people > 75, inpatients, disabled, and chronic patients have a higher risk of falling into poverty due to medical expenses.

Against the background of population aging, the prevalence of chronic diseases, hospitalization rate, and disability due to illness increase the health needs of the elderly. However, the elderly is disadvantaged in the process of obtaining resources. Therefore, it is crucial to determine the risk characteristics of this important group with physiological, social, and health vulnerability and to maximize the effectiveness of the dividend effect of welfare policies. Through our comprehensive analysis, we find that the vulnerable middle-aged and elderly people population in China is characterized by the following characteristics:

### Regional level: inter-regional macroeconomics is not the main driver for reducing the risk of health poverty

When the original poverty rate is at the same level, there is a tendency for IME to occur in the central and western regions due to the purchase of health services. The incidence of IME is significantly higher in the central and western regions than in the east, which is consistent with the distribution of poverty-stricken populations in China. Guo also demonstrated significant regional differences in the rural poverty-stricken groups and gradually gather in the central and western regions over time in China between 1978 and 2014 [47].

Inter-regional macroeconomics is not the main driver for reducing the risk of health poverty. For example, Shaanxi and Jiangsu belong to different regions but have the same poverty rate. In 2015, Shaanxi’s GDP was 180.218 billion-yuan, accounting for only a quarter of Jiangsu (70,116.38 billion yuan), but the incidence of IME (5.26%) was lower than in Jiangsu (6.13%).

Thus, the economic development-centered approach to regional poverty alleviation has not reached the maximum point of convergence with China’s current demand for health. Basu found that, in India, when poverty develops to a certain stage, the poverty reduction effect incurred by the increase in economic income would reduce under the influence of other specific factors (1951–1991) [48]. Our data can better explain that residents’ physiological characteristics and medical insurance impact a family’s IME and CHE more than the overall macroeconomic level. The indiscriminate poverty alleviation policy has reduced poverty in provinces and regions, but there are still differences in poverty caused by the use of health services.

Therefore, while paying attention to economic development and driving poverty alleviation, we should accurately target the characteristics of the poor, capture the poverty-reducing characteristics at the individual, family, and institutional levels, prioritize vulnerability, and improve the targeting accuracy of poverty alleviation policies for the poor [49].

### Individual level: the superposition of multiple health vulnerabilities exacerbated the risk of poverty among middle-aged and elderly people

High health service needs and utilization households with chronic disease and disability members, but with lower reimbursement ratio increase the difficulty for residents to obtain health rights. For example, the prevalence and hospitalization rates of respiratory disease patients were 17.4 and 16.7%, respectively, but the OOP proportion was as high as 46.7%, nearly 10% higher than for diabetic patients (37.1%). Callander calculated that Australian households with chronic disease (COPD) pay 109% more for care than those without health problems [50].Middle-aged and elderly patients with chronic diseases will increase the demand and utilization of health services as the outpatient visit rate and hospitalization rate increase. If there is a low actual reimbursement ratio and a high proportion of OOP, it will inevitably lead to a catastrophic increase in health expenditure risk. This shows that, people with chronic diseases or low social status have a heavy burden of disease due to low co-payments or no compensation [51–53]. Therefore, higher cost sharing is important for reducing the burden on residents.

The age of the head of household affects the incidence of IME. As the head of the household grows,the risk of IME increases by 1.83 percentage point. Thus, aging is one of the key factors hindering poverty reduction. Income inequality and reduced autonomy have increased the sensitivity of older groups to health rights and health equity [54]. According to the Global Burden of Disease Study, 23% of the global burden of disease occurs in older people, and chronic non-communicable diseases have a major impact on this burden [55].

Although the health vulnerability of the middle-aged and elderly people is often regarded as an intrinsic property, individuals can reduce their exposure to health rights by providing corresponding social support. More importantly, such support reduces barriers to entry for health services for the elderly, including raising the government’s high co-payments and deductibles, and strengthening the policy inclination of the elderly [56].

### Family level: families with low risk of mutual aid

Family size is negatively correlated with incidence of IME,as the member of family grows, the risk of IME is reduced by 1.5 percentage points. Connie noted that family size is related to the number of individuals entering the labor market, and the two are negatively correlated. Once the family faces unemployment, it prolongs the poverty time and poverty rate [57].

Medical insurance is less effective for low-income families. The proportion of OOP in poor households accounted for 21.5% of the household’s ability, which is 6% higher than the wealthiest households (15.1%). Furthermore, for every 1% increase in household income, the incidence of IME is reduced by 0.6%. Sujin Kim also found that after the implementation of medical insurance, the coefficient of change of the CHE rate in high-income groups (1.424) exceeded that in low-income groups (0.544); that is, the policy protection of low-income groups was less than that of high-income groups [58].

The rural poor remain the key target group for poverty alleviation. The incidence of IME in rural areas (7.72%) is higher than in urban areas (5.39%). Living in the city reduces the rate of IME by 2.8%. The hospitalized reimbursement ratio of MIUE is 68.07%, while that of the NCMS is only 37.1%, accounting for only half of the MIUR (25.0%). Urban households with higher income levels are better able to withstand the burden of health expenditures. Ye Li used China’s fourth health service survey data to calculate that the CHE of MIUE and MIUR were 9.4 and 8.5% respectively, far lower than the NCMS (14.8%) [59].

### Medical insurance level:the design of medical insurance lacks policy inclination for special populations

Participating in medical insurance increased the risk of IME by 16.5 percentage points. The protective effect of the medical insurance system has been offset by the rapid rise in medical expenses,health-care needs and service utilization. It can be seen that the medical insurance system has not played a full protective role and is likely to increase the medical burden,other scholars have reached similar conclusions. Some scholars have found that under the 30% threshold for catastrophic medical expenditures, rural residents participating in basic medical insurance will increase the risk of catastrophic medical expenditures for families by 31.8% [60]. Wagstaff et al. found that,insurance significantly increases the risks of catastrophic expenditures (at the 10% thresholds)by 42.2% [61].To explore the failure of medical insurance, we analyzed the top five factors affecting different medical insurances and found that chronic patients, inpatients, disabled people, and people over 65 are the key populations in need of poverty alleviation. The specific reasons for failure are as follows:

#### The medical insurance does not impose policy inclinations on the middle-aged and elderly people groups with high health service demand and utilization, but only reduces the access standards of this group

According to our data, the overall hospitalization reimbursement ratio for the elderly over 45 years old is 49.7%, which is lower than the. European Commission (EU)‘s standard for the reimbursement of major illnesses of not less than 60% [62]. In the case of cancer patients, the hospitalization reimbursement ratio for cancer patients is only 57.4%. Goss calculated the cancer costs in different countries and found that the proportion of household expenses for patients in the US was only 20.9%, while in China it was as high as 78.8% [63]. Because it is difficult to design access mechanisms for major diseases that cover all chronically ill patients with large medical expenses, MIUE and MIUR with higher income levels are also suffering from IME, and poverty is as high as 25%. Simultaneously, the difference in reimbursement ratio for chronic diseases caused by medical insurance introduces risks within the system, which makes the reimbursement of mental illness patients (53.9%) of MIUE nearly 28.4% lower than those with low-grade diabetes (81.5%). Some chronic drugs are not included in the reimbursement range, which increases patients’ OOP expenses. Meanwhile, the patient’s transportation expenses, nursing expenses, lost time, and preventive health care expenses are not within the scope of reimbursement, which greatly increases the household’s disease burden.

#### The difference between the type and internal design of the medical insurance leads to a certain gap between different income groups. There is a lack of policy inclination for low-income groups, while high income groups have a more advantageous compensation level by medical insurance

First, the difference between the medical insurances leads to different risks of IME. According to our data, the NCMS has a high incidence of IME, reaching 7.9%, approximately 2.42 times that of MIUE. Although medical insurance integration has been implemented in some places to alleviate the huge differences between groups, the incidence of IME is the highest (8.5%), at 0.6 percent higher than the NCMS. Therefore, it has not been possible for residents to achieve equal access to healthcare services. Su, Min reached the same conclusion [64]. The fundamental reason for this lack of equality is the low financing level, combined with a reimbursement package that does not consider the traffic, economic level, and family-mutual aid capabilities.

Additionally, the internal system design of medical insurance lacks economic support for low-income families and protects the rich more. In our study, the sub-poor group had a hospitalization reimbursement ratio of only 42%, lower than the highest at 50.5%. A similar phenomenon occurred in India. With only government expenditure, the poorest households receive only 10% of medical care, while the wealthiest families receive up to 33% of social subsidies [65].

#### The medical insurance system has not yet transformed regional poverty alleviation into individual alleviation. Therefore, it fails to accurately target vulnerable groups, and there are weak links in the connection with the existing poverty alleviation related systems

China’s health poverty alleviation system is a muti-path health protection system that integrates a basic medical insurance scheme, major illness insurance scheme, medical assistance scheme, and disease emergency assistance scheme. It aims to improve the health protection level of poverty-stricken areas and poor people. However, examining our results, chronic patients, the elderly, and other groups have increased the risk of IME after using health services. Thus, the health insurance still has a long way to go to improve health poverty alleviation. Firstly, since 7.4% of the middle-aged and elderly people are unable to enjoy health insurance rights, we should adhere to the basic guarantee function of the basic medical insurance for the over 45 age-group, and create a guarantee for vulnerable and poverty-stricken groups. Secondly, the poverty line should be changed before accurately measuring the poverty levels of different age-groups, incomes, and special needs through quantitative analysis methods. The government should improve the medical assistance policies for major diseases, and consider the poverty-stricken population and personal medical economic burden on the basis of main classification. Thirdly, the effective connection between the basic medical insurance scheme and major disease insurance scheme and medical assistance system should be strengthened. Participation in basic medical insurance should be enforced for the elderly over 65 years old, mental illness, cancer, diabetes, inpatients, and disabled people who are high-risk groups for IME. They should also participate voluntarily in the supplementary medical insurance for major illnesses. Insured persons who are hospitalized due to illness meet the scope of payment for the major illness supplementary medical insurance fund. Moreover, to provide reimbursement, a payment line should be established, with proportional segmentation, cumulative calculation, and the highest capping method.

## Conclusions

The original poverty promoting policies has not reached the maximum point of convergence with China’s current demand for health. The overlapped health vulnerabilities exacerbated the risk of poverty among the middle-aged and elderly people and households with high health needs and utilization. In addition, the medical insurance have proven to be insufficient for protection against economic burden of poor households. So, special health needs, age, and household capacity to pay should be comprehensively considered while strengthening the connection between the basic insurance with supplementary insurance.

## Data Availability

Dataset available from the CHARLS repository, http://charls.pku.edu.cn.

## Abbreviations

CHE: Catastrophic Health Expenditure
IME: Impoverishment by Medical Expenses
MIUE: Urban employees’ Insurance Medical
MIUR: Urban resident Insurance Medical
NCMS: New rural Cooperative Medical System
PL: Poverty Line
SE: Household subsistence expenditure
CTPA: Household’s capacity to pay
OOP: Out-of-pocket health expenditure
EXP: Household consumption expenditure
IV: Instrumental Variable

## References

[1] Asadullah MN, Savoia A (2018). Poverty reduction during 1990–2013: did, millennium, development goals adoption and state capacity matter?. World Dev.

[2] World Bank. Poverty Overview.2019. http://www.wordbank.org/en/topic/poverty/overview. Accessed April 03, 2019.

[3] Alkire S, Conconi A, Robles G. The global multidimensional poverty index (MPI): 5-year methodological note. The Oxford Poverty and Human Development Initiative (OPHI), Oxford Department of International Development, University of Oxford.2016.

[4] Hotez PJ, Thompson TG (2009). Waging peace through neglected tropical disease control: a US foreign policy for the bottom billion. Plos Neglect Trop D.

[5] Savadogo G, Souarès A, Sié A, Parmar D, Bibeau G, Sauerborn R (2015). Using a community-based definition of poverty for targeting poor households for premium subsidies in the context of a community health insurance in Burkina Faso. BMC Public Health.

[6] Alkire S, Foster J (2011). Counting and multidimensional poverty measurement. J Public Econ.

[7] The State Council Leading Group Office for Poverty Alleviation and Development. China provides Chinese wisdom for global poverty reduction and South-South cooperation.2019. http://www.cpad.gov.cn/art/2019/7/12/art_136_99964.html. Accessed July 12, 2019.

[8] Teerawichitchainan B, Knodel J. Long-term care needs in the context of poverty and population aging: the case of older persons in Myanmar. J Cross Cult Gerontol. 2018;33(2):143–62. 10.1007/s10823-017-9336-2 28988375

[9] Chen SX, Li JJ, Lu SF, Xiong B (2017). Escaping from poverty trap: a choice between government transfer payments and public services. Glob Health Res Policy.

[10] State Council Poverty Alleviation Office. Three batches of action plan for health poverty alleviation project.2017.http://www.cpad.gov.cn/art/2017/10/ 11/art_2164_72003.html. Accessed October 17, 2017.

[11] National Health and Family Planning Commission of the People’s Republic of China. Progress in disease prevention and control in China.2015. http://www.nhc.gov.cn/wjw/gfxwj/201304/b8de7b7415ca4996b3567e5a09e43300shtml.Accessed May 8, 2012.

[12] National Health and Family Planning Commission Statistical Information Center. The fifth National Health Service Survey and analysis report of the People’s republic of China, 2013. China Union Medical University Press, 2015.

[13] Li Y, Wu QH, Liu CJ, Kang Z, Xie X, Yin H, Jiao ML, Liu GX, Hao YH, Ning N (2014). Catastrophic Health Expenditure and Rural Household Impoverishment in China: What Role Does the New Cooperative Health Insurance Scheme Play?. Plos One.

[14] Liu XY, Sun XJ, Zhao Y, Meng QY (2016). Financial protection of rural health insurance for patients with hypertension and diabetes: repeated cross-sectional surveys in rural China. BMC Health Serv Res.

[15] National Health and Family Planning Commission of China. Statistical yearbook of health and family planning in China.2016 .

[16] World Bank. Tracking universal health coverage: 2017 global monitoring report. 2017. Available at: http://www.worldbank.org/en/topic. Accessed March 29, 2018.

[17] National Health Commission of the People's Republic of China. In 2017, the national public hospitals realized the cancellation of new and old system conversion drug additions.2018.http://www.nhc.gov.cn/wjw/xgbd/201802/d19e64a17226441c80812a48e8211452.shtml.Accessed February 13, 2018.

[18] Kumar K, Singh A, Kumar S, Ram F, Singh A, Ram U, Negin J, Lowal P. Socio-economic differentials in impoverishment effects of out-of-pocket health expenditure in China and India: evidence from WHO SAGE. PLoS One. 2015;10. 10.1371/journal.pone.0135051 PMC4536075 26270049

[19] Yang Y, John W, Shen C (2010). Social security for China's rural aged: a proposal based on a universal non-contributory pension. Int J Soc Welf.

[20] F. Cai, J. Giles, P. Keefe, D. Wang. The elderly and old age support in Rural China: challenges and prospects. World Bank, Washington (2012).

[21] Kumar S (2000). Economists advocate health improvement to reduce poverty. Lancet.

[22] Guidance on the implementation of health poverty alleviation projects. The State Council The People’s Republic of China.http://www.gov.cn/xinwen/2016-06/21/content_5084195.htm.Accessed:June 21.2016.

[23] Research for universal health coverage: World health report 2013. World Health Organization. https://www.who.int/whr/2013/report/en/. Accessed Aug 2013.

[24] Healthy China Project.Yan Xiaozheng, the founder of China Health Poverty Alleviation Project and director of Health China Project Management Committee. http://www.jkzggc.com/detail-64.shtml. Accessed 22 June 2019.

[25] The State Council Leading Group Office of Poverty alleviation and Development. Letter on Reply to Proposal No. 0062 .http://www.cpad.gov.cn/art/2018/12/24/art_2203_92372.html Accessed December 24, 2018.

[26] National Health Committee of the People's Republic of China. Guidance on the implementation of health poverty alleviation projects.http://www.nhc.gov.cn/jkfpwlz/fpzllist/201606/d16de85e75644074843142dbc207f65d.shtml. Accessed June 21, 2016.

[27] Hsieh CR, Qin X. Depression hurts, depression costs: the medical spending attributable to depression and depressive symptoms in China [J]. Health Econ. 2017. 10.1002/hec.3604 28990318

[28] National Health Commission of the People's Republic of China. Notice on Issuing the National Essential Medicines List (2018 Edition).http://www.nhc.gov.cn/yaozs/s7656/201810/c18533e22a3940d08d996b588d941631.shtml. Accessed October 25, 2018.

[29] The Central People's Government of the People's Republic of China. The 12th Five-Year Plan and Implementation Plan for Deepening the Reform of Medical and Health System.http://www.gov.cn/zhengce/content/2012-03/21/content_6094.htm Accessed March 14, 2012.

[30] Yi C, Julie S, Castiel CZ (2019). Income-dependent impacts of health insurance on medical expenditures: Theory and evidence from China [J]. China Econ Rev.

[31] Tian Y, Yi J, Hu XM, Li YP, Zhang LD, Wan D, Jia SL (2019). China’s health policies for poverty alleviation in the field of medical security system: an analysis of status quo.[J]. Chinese Rural Health Serv Adm.

[32] Xu K. Distribution of health payments and catastrophic expenditures methodology. Geneva: Department of Health System Financing.”*World Health Organization.* 2005.

[33] Wooldridge JM (2004). Further results on instrumental variables estimation of average treatment effects in the correlated random coefficient model. Econ Lett.

[34] Jelena A, Milena P, Bernd R, Wim G (2016). Catastrophic Health care expenditure among older people with chronic diseases in 15 European countries. PLoS One.

[35] Lewbel A (2007). Endogenous selection or treatment model estimation. J Econ.

[36] Wang XW. A study on the impact of Health on the labor supply behavior of the mid-aged and elderly of China. *Anhui University of Finance and Economics* 2016.

[37] Xu XD, Wu WQ (2015). Analysis on influencing factors of primary health care choice in middle-aged and elderly population based on CHARLS data. Chinese J Health Policy.

[38] Hu HW, Zhang L, Li JY, Du YX, Wang JR (2015). Does urban residents basic medical insurance worsen the medical burden to the elderly?-system evaluation with household medical burden. Chinese Sci Res Aging.

[39] Gu NY, Gai Y, Hay JW. The effect of patient satisfaction with pharmacist consultation on medication adherence: An instrumental variable approach. Pharmacy Practice. 2008;6(4). 10.4321/s1886-36552008000400006 PMC4141731 25157295

[40] Hall AR, Rudebusch GD, Wilcox DW (1996). Judging instrument relevance in instrumental variables estimation. Inter Econ Review.

[41] Ma R, Huang L, Zhao D, Xu L (2014). China critical illness insurance policy-the recent developments and prospects. Value Health.

[42] Mestrum F (2003). Poverty reduction and sustainable development. Environ Dev Sustain.

[43] Bulletin of the National Health and Family Planning Commission of the People's Republic of China. Guidance on the implementation of health poverty alleviation projects. 2016 (6): 243–248.

[44] Amaya-Lara LJ (2016). Catastrophic expenditure due to out-of-pocket health payments and its determinants in Colombian households [J]. Inter J for Equity in Health.

[45] Brinda, Ethel Mary et al. "Health service use, out-of-pocket payments and catastrophic health expenditure among older people in India: The WHO Study on global AGEing and adult health (SAGE)." J Epidemiol Commun H*.* 2015(69.5):489–494.at: https://www.researchgate.net/publication/2707057. 10.1136/jech-2014-204960 25576563

[46] Ghiasvand H, Gorji HA, Maleki M, et al. Catastrophic Health Expenditure Among Iranian Rural and Urban Households, 2013–2014[J]. Iran Red Crescent Me. 2015;17(9). 10.5812/ircmj.30974 PMC4601211 26473081

[47] Guo YZ, Zhou Y, Liu YS. Targeted poverty alleviation and its practices in rural China: a case study of Fuping county, Hebei Province. J Rural Stud. 2019:0743–167.

[48] Basu S, Mallick S (2008). When does growth trickle down to the poor? The Indian case. Camb J Econ.

[49] Liu YS, Liu JL, Zhou YJ. Spatio-temporal patterns of rural poverty in China and targeted poverty alleviation strategies. J Rural Stud. 2017:66–75.

[50] Callander EJ, Corscadden L, Levesque JF (2017). Out-of-pocket healthcare expenditure and chronic disease – do Australians forgo care because of the cost?. Aust J Prim Health.

[51] Sajad V, Aziz R, Farzad F K, Firooz E, Javad J, Abdollah A, Abbas G. Decomposition of Socioeconomic Inequality in Catastrophic Health Expenditure: An Evidence from Iran, Clinical Epidemiology and Global Health,2019,ISSN 2213-3984.

[52] Yardim MS, Cilingiroglu N, Yardim N (2010). Catastrophic health expenditure and impoverishment in Turkey [J]. Health Policy.

[53] Lee JE, Shin HI, Do YK, et al. Catastrophic Health Expenditures for Households with Disabled Members: Evidence from the Korean Health Panel [J]. J Korean Med Sci. 2016;31(3). 10.3346/jkms.2016.31.3.336 PMC4779856 26955233

[54] Acciai F (2018). The age pattern of social inequalities in health at older ages: are common measures of socio-economic status interchangeable?. Public Health.

[55] World Health Organization. Health in 2015: from MDGs to SDGs. 2015. Available at: https://www.who.int/gho/publications/mdgs-sdgs/en/.Accessed December 2015.

[56] Ye L, Shia BC, Fang Y, Lee TS (2019). Heterogeneous health profiles and healthcare utilization of the middle-aged and elderly with multiple health insurance schemes in China. Public Health.

[57] Bayudan-Dacuycuy C, Lim JA (2013). Family size, household shocks and chronic and transient poverty in the Philippines. J Asian Econ.

[58] Kim S, Kwon S (2015). Impact of the policy of expanding benefit coverage for cancer patients on catastrophic health expenditure across different income groups in South Korea. Soc Sci Med.

[59] Li Y, Wu QH, Xu L, Legg D, Hao YH, Gao LJ, Ning N, Wan G (2012). Factors affecting catastrophic health expenditure and impoverishment from medical expenses in China: policy implications of universal health insurance. B World Health Organ.

[60] Wang YQ, Xu QT (2019). Can basic medical insurance reduce catastrophic Health spending for residents? Evidence from data of CHARLS [J]. Financ Theory Pract.

[61] Wagstaff A, Lindelow M (2008). Can insurance increase financial risk?: the curious case of health insurance in China [J]. J Health Econ.

[62] European Commission.https://www.ec.europa.eu/eurostat/en/web/products-statistical-working-papers/Accessed March 26,2014.

[63] Goss PE, Strasser-Weippl K, Lee-Bychkovsky BL, Fan L, Li JJ, Chavarri-Guerra Y, Liedke PE, Pramesh CS, Badovinac-Crnjevic T, Sheikine Y, Chen Z, Qiao YL, Shao ZM, Wu YL, Fan DM, Chow LW, Wang J, Zhang Q, Yu SY, Shen G, He J, Purushotham A, Sullivan R, Badwe R, Banavali SD, Nair R, Kumar L, Parikh P, Subramanian S, Chaturvedi P, Iyer S, Shastri SS, Digumarti R, Soto-Perez-de-Celis E, Adilbay D, Semiglazov V, Orlov S, Kaidarova D, Tsimafeyeu I, Atishchev S, Danishevskiy KD, Hurlbert M, Vail C, St Louis J, Chan A (2014). Challenges to effective cancer control in China, India, and Russia [J]. Lancet Oncol.

[64] Su M, Si YF, Zhou ZL (2017). The effects of China's three basic health insurance schemes on the equity of health-related quality of life: a cross-sectional survey using coarsened exact matching. Lancet.

[65] Garg P, Nagpal J (2014). A review of literature to understand the complexity of equity, ethics and management for achieving public health goals in India. J Clinical Diag Res.

